# Auger-Emitting Radionuclides in Radiopharmaceutical Research: Decay-Associated Processes, Vector-Dependent Localization, and Translational Perspectives

**DOI:** 10.3390/ijms27146445

**Published:** 2026-07-20

**Authors:** Klaus Schomäcker, Ferdinand Sudbrock, Melanie Freifrau von Brandenstein, Baki Akgül, Martin Hufbauer, Thomas Fischer, Sabri E. M. Sahnoun, Felix Dietlein, Philipp Krapf, Markus Dietlein, Alexander Drzezga

**Affiliations:** 1 Department of Nuclear Medicine, Faculty of Medicine and University Hospital Cologne, University of Cologne, Kerpener Str. 62, 50937 Cologne, Germany; ferdinand.sudbrock@uk-koeln.de (F.S.); thomas.fischer@uk-koeln.de (T.F.); p.krapf@fz-juelich.de (P.K.); markus.dietlein@uk-koeln.de (M.D.); alexander.drzezga@uk-koeln.de (A.D.); 2 Department of Urology, Faculty of Medicine and University Hospital of Cologne, University of Cologne, 50937 Cologne, Germany; melanie.freifrau-von-brandenstein@uk-koeln.de; 3 Institute of Virology, Faculty of Medicine and University Hospital of Cologne, University of Cologne, 50935 Cologne, Germany; baki.akguel@uk-koeln.de (B.A.); martin.hufbauer@uk-koeln.de (M.H.); 4 Computational Health Informatics Program, Boston Children’s Hospital, Harvard Medical School, Boston, MA 02115, USA; felix.dietlein@childrens.harvard.edu; 5 Nuclear Chemistry (INM-5), Institute of Neuroscience and Medicine, Forschungszentrum Jülich GmbH, Wilhelm-Johnen-Straße, 52428 Jülich, Germany; 6 German Center for Neurodegenerative Diseases (DZNE), Bonn-Cologne, Venusberg-Campus 1/99, 53127 Bonn, Germany

**Keywords:** Auger electrons, internal conversion electrons, Coster–Kronig electrons, Coulomb explosion, cellular dosimetry, subcellular localization, DNA damage, radiopharmaceutical therapy, technetium-99m, terbium-161

## Abstract

Auger-electron-emitting radionuclides offer a distinctive route toward molecular-scale radiotherapy because their biological effects depend primarily on the nanoscale location of the decay event rather than on long-range tissue penetration. This review examines Auger-emitting radionuclides from a radiopharmaceutical perspective, integrating decay-associated physicochemical processes, cellular dosimetry, subcellular targeting, and translational relevance. We first discuss the physical determinants of Auger radiotoxicity, including Auger and Coster–Kronig electrons, internal-conversion electrons, local ionization density, and possible molecular consequences of highly localized Auger cascades. Cellular S values are used to illustrate how the same radionuclide can produce markedly different absorbed doses depending on whether activity is localized in the nucleus, cytoplasm, cell membrane, or neighboring cells. Iodine-125 incorporated into DNA as [^125^I]iododeoxyuridine ([^125^I]IUdR) remains the classical reference model for maximal Auger-mediated radiotoxicity. However, this direct DNA-incorporation model should not be generalized to receptor-targeted radiopharmaceuticals, which usually achieve indirect nuclear, chromatin-associated, perinuclear, membrane-associated, or vesicular localization rather than incorporation into DNA. These alternative source–target geometries may also produce biologically relevant effects, but they are mechanistically distinct from DNA-proximal [^125^I]IUdR decay. Particular attention is given to iodine-123 versus iodine-125, the influence of physical half-life and specific activity, the interpretation of terbium-161 as a hybrid β^−^/internal-conversion/Auger emitter, and the overlooked radiobiological relevance of diagnostic Auger emitters such as technetium-99m, indium-111, and gallium-67. We conclude that Auger-emitter radiopharmaceuticals cannot be ranked by electron yield or decay scheme alone. Their therapeutic or toxicological relevance emerges from the integration of decay-associated physicochemical processes, cellular source–target dosimetry, intracellular trafficking, retention, and the temporal realization of dose delivery.

## 1. Introduction

Theranostics has become a defining concept in contemporary nuclear medicine. It denotes the use of molecularly matched diagnostic and therapeutic radiopharmaceuticals that address the same disease-associated target and thereby integrate target visualization, patient selection, dosimetry, and radionuclide therapy [1]. Clinically established and emerging examples include prostate-specific membrane antigen (PSMA)-targeted urea-based ligands, somatostatin receptor-directed agents, HER2-directed approaches, and receptor-targeted strategies involving steroid hormone receptors such as the estrogen receptor (ER) [2,3,4,5,6,7,8,9,10,11].

The Auger effect is an atomic de-excitation process that follows inner-shell ionization, most commonly resulting from electron capture, internal conversion, or photoionization. The resulting vacancy initiates a cascade of electronic rearrangements that may produce the emission of one or more Auger electrons. Importantly, the biological significance of this process does not reside in a single emitted particle, but in the highly localized energy release generated by the cascade as a whole [12,13]. In addition to electron emission, the Auger cascade may transiently leave the daughter atom or molecular site in a highly ionized state; the subsequent charge redistribution, neutralization, and possible Coulomb-driven fragmentation have been discussed as additional contributors to molecular damage, although their quantitative radiobiological relevance remains incompletely defined [14,15].

Because the emitted electrons possess very low energies, their range in biological matter is extremely limited, typically spanning nanometers to a few micrometers. Consequently, the biological outcome of a radionuclide decay followed by Auger-electron emission depends critically on the exact intracellular location of the radionuclide at the moment of decay. When this process occurs in immediate proximity to nuclear DNA, the resulting cascade can deposit substantial energy within a molecular volume comparable to that of the DNA double helix, producing highly clustered and poorly repairable lesions [12,16,17,18].

The distinctive opportunity offered by nuclear medicine lies in its ability to combine radioactive decay with molecular targeting. Radiopharmaceuticals can be engineered to recognize tumor-associated structures such as cell-surface receptors or transporters. Following receptor binding, many ligands undergo internalization and intracellular trafficking, thereby transporting the radionuclide into the cellular interior [19].

In the case of Auger-emitting radionuclides, this biological transport becomes decisive. Because the radiotoxic action of the Auger cascade is confined to nanometer-scale distances, the therapeutic effect depends on whether radioactive decay occurs in the vicinity of critical molecular targets, above all nuclear DNA. Although direct DNA incorporation or DNA-proximal nuclear localization provides the most favorable source–target geometry for highly localized dose deposition, Auger electron-induced cytotoxicity is not confined to nuclear targeting. Membrane-associated damage, ROS-mediated pathways, local cross-dose effects, and bystander mechanisms may also contribute to biological efficacy [12,19,20,21].

In this sense, the radiopharmaceutical acts as a molecular “Trojan horse”, selectively guiding the radionuclide toward malignant cells and potentially positioning the decay event near the cell nucleus. The therapeutic potential of Auger-based radiopharmaceuticals therefore arises from the interplay between atomic physics and molecular targeting.

Accordingly, Auger-emitting radionuclides introduce the concept of molecular-scale radiotherapy, in which the biological impact of radiation is determined not primarily by tissue range, but by the precise intracellular positioning of radioactive decay. In therapeutic applications, however, this concept remains largely translational and requires radiopharmaceutical designs that bring the decay event into close spatial proximity to critical intracellular targets.

This review examines Auger-emitting radionuclides from a radiopharmaceutical perspective, focusing on their physical determinants, cellular source–target dosimetry, temporal dose realization, molecular targeting requirements, intracellular delivery, and translational implications for both therapeutic and diagnostic radiopharmaceuticals.

## 2. Physical Determinants of Auger-Electron Radiotoxicity

As outlined above, Auger-electron emission follows inner-shell ionization and the subsequent electronic relaxation cascade. Depending on the initiating process and atomic structure, this cascade may generate Auger, Coster–Kronig, super-Coster–Kronig, and internal-conversion electrons, together with characteristic X-rays and secondary low-energy electrons. Radiative relaxation by emission of characteristic X-rays and, to a lesser extent, ultraviolet photons represents an important competing pathway to non-radiative Auger and Coster–Kronig emission.

This competition is governed by shell-specific fluorescence yields and becomes increasingly relevant for higher-Z elements, where characteristic X-ray emission may dominate substantial parts of the atomic relaxation cascade. Because many of the emitted electrons have very short ranges in condensed biological matter, the resulting local energy deposition can be confined to nanometer dimensions [22].

Short-range electron emission and the resulting highly localized energy deposition constitute the established physical basis of Auger radiobiology. In addition, the relaxation cascade may generate highly charged and electronically excited molecular states, promote charge redistribution, and contribute to Coulomb-driven fragmentation of the labeled compound or adjacent biomolecules [23]. In condensed-phase environments, additional non-local relaxation processes may also occur. These include interatomic or intermolecular Coulombic decay (ICD) and electron-transfer-mediated decay (ETMD), which can generate low-energy electrons and reactive species in neighboring molecules. However, their quantitative contribution to DNA damage, cellular toxicity, and other radiobiological endpoints under realistic intracellular conditions remains incompletely defined [24,25,26].

Thus, an Auger cascade may be conceptualized as generating a highly localized physicochemical reaction volume around the decay site. Within this reaction volume, low-energy electrons can induce ionization, excitation, and dissociative electron attachment, while subsequent chemical processes may lead to radical formation and molecular fragmentation. If the decay occurs within nanometer distance of chromatin, short-range electron energy deposition and the resulting local chemical reactions may produce clustered DNA lesions that are difficult to repair [17,27,28].

These considerations explain why the therapeutic potential of Auger-emitting radionuclides depends not only on the number of emitted electrons, but also on their energy distribution, spatial confinement, and the subcellular position of the decay event. Additional decay-associated physicochemical processes may further modify local molecular damage, although their quantitative contribution to radiobiological outcome remains uncertain. A comparative assessment of Auger emitters therefore requires a distinction between biological delivery and the intrinsic per-decay dose-deposition potential of a radionuclide under defined source–target assumptions. The following sections develop this distinction and introduce a relative framework for comparing the physical dose-deposition characteristics of different Auger-emitting radionuclides.

### 2.1. Concept of Intrinsic Per-Decay Dose-Deposition Potential

In targeted radionuclide therapy, the final biological outcome reflects the interplay between target recognition, tumor accumulation, cellular uptake, subcellular localization, and the physical decay process. The biological component determines whether the radioligand reaches the tumor cell, binds to its cognate molecular structure, and occupies a therapeutically relevant subcellular compartment at the time of decay, whereas the physical decay process determines the local pattern of ionization and excitation once radioactive decay occurs.

For Auger-electron emitters, this distinction is particularly important. If subcellular localization is idealized as equivalent—for example, decay in immediate proximity to nuclear DNA—the comparison between radionuclides becomes predominantly physical: how much densely localized energy can a single decay concentrate within a molecular volume?

We therefore define intrinsic per-decay dose-deposition potential as a comparative physical and dosimetric descriptor of the energy deposited by an individual radionuclide decay under equivalent source–target assumptions, independent of target recognition, tumor accumulation, cellular uptake, and radiopharmaceutical delivery. This descriptor does not represent radiotoxicity or biological effectiveness and should not be interpreted as a direct measure of relative biological effectiveness, clonogenic survival, DNA double-strand break yield, or therapeutic efficacy.

This descriptor provides a framework for comparing Auger-emitting radionuclides under equivalent source–target and localization assumptions. For this purpose, iodine-125 serves as a reference radionuclide because its Auger cascade and its high radiobiological potency after DNA incorporation are well characterized [15,18].

### 2.2. From Intrinsic Per-Decay Dose-Deposition Potential to Cellular Dose: Limitations of Simple Ranking Approaches

A comparison of Auger-electron-emitting radionuclides cannot be reduced to the number of emitted Auger electrons, the mean electron energy, or a single radionuclide-specific “quality factor”. This is particularly important because the biological relevance of short-range electrons depends not only on their number per decay, but also on their individual energy distribution. Electron energy determines range, local ionization density, and the spatial relationship between the decay site and the radiosensitive target. Accordingly, the biological effect of an Auger-emitting radionuclide emerges from the combination of its emission spectrum, the source–target geometry at the cellular and subcellular level, and the radiosensitive structure reached by the decay event. Therefore, any ranking of Auger emitters must distinguish between intrinsic per-decay dose-deposition potential and biologically realized dose deposition.

A useful conceptual basis is provided by the cellular S-value approach developed by Goddu, Howell, and co-workers [29,30]. In this framework, absorbed dose is calculated for defined source and target regions, such as nucleus-to-nucleus, cytoplasm-to-nucleus, or cell-surface-to-nucleus configurations. Cellular S values quantify absorbed dose per unit cumulated activity for explicitly defined source and target regions. They are geometry-dependent dosimetric quantities and are not direct surrogates for radiotoxicity, relative biological effectiveness, clonogenic survival, DNA double-strand break induction, or therapeutic efficacy. This approach makes explicit that the same radionuclide may produce very different nuclear doses depending on whether it is localized in the nucleus, cytoplasm, cell membrane, or neighboring cells. Thus, the relevant quantity is not the radionuclide alone, but the source–target relationship in which its decay takes place.

To illustrate this relationship, Table 1 combines physical emission characteristics with cellular S values for selected Auger-electron-emitting radionuclides and β^−^-emitting comparator radionuclides. The physical columns summarize half-life, main decay mode, and the mean number and total emitted energy of Auger/Coster–Kronig and internal-conversion electrons per decay. The dosimetric columns report cellular S values in a defined spherical cell geometry according to the Goddu/Howell source–target framework [30]. Thus, the table links decay-specific emission characteristics with their consequences for absorbed dose under defined cellular source–target assumptions.

Iodine-125 is used as the reference radionuclide for the relative nucleus-to-nucleus comparison because it represents the classical benchmark for DNA-proximal Auger radiotoxicity. Accordingly, Srel(N←N) vs. I-125 denotes the nucleus-to-nucleus S value of each radionuclide normalized to the corresponding S(N←N) value of I-125. This normalization provides a common physical reference under an equivalent nuclear source–target assumption, but it should not be interpreted as a direct ranking of therapeutic efficacy. These S values are geometry-dependent total-electron dose estimates for the specified cell model and should not be interpreted as isolated Auger/Coster–Kronig, internal-conversion, or β^−^ contributions, nor as measures of biological effectiveness. Iodine-131 and lutetium-177 are included as β^−^-emitting comparator radionuclides, whereas terbium-161 is included with Goddu/Howell-analog estimates derived from published CELLDOSE data and is therefore discussed separately below [31].

Iodine-125 remains the classical reference case for a DNA-proximal Auger emitter, particularly when incorporated into DNA as iododeoxyuridine [17,18,32,33,34]. Under these conditions, the Auger cascade occurs within nanometer distance of the DNA backbone and can generate highly clustered lesions within a molecularly confined volume. If the same decay occurs outside the nucleus or at larger distances from chromatin, the biological effect is substantially reduced. Iodine-125 therefore illustrates the central principle of Auger therapy: the Auger effect becomes maximally relevant only when the decay event is positioned at, or very close to, a critical molecular target. This distinction is particularly important for terbium-161, which is not included in the iodine-125-normalized Goddu/Howell comparison shown in Table 1. Unlike the radionuclides listed there, terbium-161 is not part of the original Goddu/Howell cellular S-value dataset used for this reference comparison. It is therefore discussed separately as a β^−^/internal-conversion/Auger hybrid radionuclide, for which the most relevant comparison is not with DNA-incorporated iodine-125, but with lutetium-177 in receptor-targeted radiopharmaceutical therapy.

### 2.3. Terbium-161 as a β^−^/Conversion-Electron/Auger Hybrid Radionuclide

Terbium-161 is increasingly discussed as an Auger-electron-emitting therapeutic radionuclide and is frequently compared with lutetium-177 in targeted radionuclide therapy. However, terbium-161 should not be interpreted as a functional equivalent of DNA-localized iodine-125. Its decay profile combines β^−^ emission with internal-conversion and Auger/Coster–Kronig electrons, resulting in a hybrid radiation pattern rather than a purely DNA-proximal Auger mechanism [31,35,36].

This distinction is important for interpreting cellular and subcellular dosimetry. Terbium-161 is usually delivered as a radiometal chelate coupled to peptides, antibodies, or other targeting vectors. Its potential advantage over lutetium-177 should therefore be understood as an additional local electron-dose contribution superimposed on β^−^ irradiation, provided that the radiopharmaceutical reaches biologically vulnerable subcellular sites such as the cell membrane, endosomal or cytoplasmic compartments, perinuclear regions, or, in selected cases, the nucleus. In contrast to DNA-incorporated [^125^I]IUdR, direct incorporation into nuclear DNA is not the defining requirement for biological relevance, although nuclear or DNA-proximal localization would be expected to maximize radiotoxicity. Consequently, terbium-161 should be treated separately in comparative discussions of Auger-emitting radionuclides, and reported dose estimates should be interpreted according to whether they refer to Auger-specific or total-electron dose deposition.

#### CELLDOSE-Derived Total-Electron Dose Estimates for Terbium-161

Published Goddu/Howell cellular S values are not available for terbium-161 in the original dataset used for the iodine-125-normalized comparison in Table 1. Terbium-161 therefore requires separate consideration. Alcocer-Ávila et al. [31] reported CELLDOSE simulations comparing terbium-161 and lutetium-177 in a defined spherical cell model, using single tumor cells and small cell clusters. In the single-cell model, the cell diameter was 14 µm and the nuclear diameter was 10 µm; activity was localized at the cell surface, in the cytoplasm, in the nucleus, or uniformly within the whole cell, and the absorbed dose to the nucleus was used as the principal dosimetric endpoint.

The central result was that terbium-161 delivered higher nucleus-absorbed doses than lutetium-177 across all investigated source configurations in both single cells and small cell clusters. Depending on source localization and geometry, the increase was approximately two- to fourfold, with the largest advantage observed for intranuclear localization and smaller, but still substantial, increases for cytoplasmic and cell-surface activity [31].

This increase in nuclear absorbed dose should not be interpreted as a marginal effect. It reflects the substantially richer short-range electron component of terbium-161 compared with lutetium-177, including internal-conversion, Auger, and Coster–Kronig electrons. Thus, the separate treatment of terbium-161 in this review is not intended to minimize its relevance as a short-range electron emitter. Rather, it is meant to avoid an inappropriate direct comparison with DNA-incorporated iodine-125. In radiopharmaceutical applications, terbium-161 is best understood as a β^−^/internal-conversion/Auger hybrid radionuclide whose potential advantage over lutetium-177 arises from additional local electron dose within defined vector-dependent cellular and subcellular geometries.

For orientation, these CELLDOSE results can be related to the normalization used by Alcocer-Ávila et al. [31]. The simulations were normalized to a total released electron energy of 1436 MeV per cell. Using the reported total electron energy of 202.5 keV per decay for terbium-161, this normalization corresponds to approximately 7.1 × 10^3^ nuclear transformations. The reported absorbed doses can therefore be expressed as approximate dose-per-transformation values for the specific CELLDOSE geometry. This conversion is useful within a cellular dosimetry framework, but the resulting values represent total-electron dose estimates, including β^−^, internal-conversion, and Auger/Coster–Kronig electron contributions.

Accordingly, these data are most informative for the specific comparison between terbium-161 and lutetium-177. In this setting, terbium-161 should be interpreted as a lutetium-177 analogue with an enhanced local electron-dose component, rather than as a classical iodine-125-type Auger emitter. The higher nuclear doses reported for terbium-161 reflect the combined contribution of β^−^ particles, internal-conversion electrons, and Auger/Coster–Kronig electrons within small-cell geometries. For this reason, these values should not be incorporated into the iodine-125-normalized Goddu/Howell ranking shown in Table 1. Instead, they support a separate interpretation: terbium-161 may provide a cellular and subcellular dose advantage over lutetium-177, but the biological relevance of this advantage remains dependent on the vector-mediated distribution and residence of the radiopharmaceutical at vulnerable subcellular sites.

### 2.4. Physical Half-Life, Specific Activity, and Biologically Deliverable Activity

Beyond decay-specific emission properties and cellular S values, the physical half-life of a radionuclide influences how much activity can, in principle, be associated with a given amount of radiolabeled compound. For a carrier-free or no-carrier-added preparation, the theoretical molar activity is inversely proportional to the physical half-life. Thus, shorter-lived radionuclides can provide higher theoretical molar activities than longer-lived radionuclides, provided that nonradioactive carrier does not substantially dilute the labeled product. For iodine-123 and iodine-125, this relationship can be approximated as:

$$\frac{A_{m}(iodine-125)}{A_{m}(iodine-123)}=\frac{T_{1/2}(iodine-125)}{T_{1/2}(iodine-123)}=\frac{59.4d}{13.2h}=\frac{1425.6h}{13.2h}\approx 108$$

Thus, under ideal carrier-free conditions, iodine-123 could theoretically provide molar activities approximately 100-fold higher than iodine-125. The practically achieved molar activity, however, also depends on radionuclide production, radionuclidic and isotopic purity, chemical processing, precursor mass, labeling efficiency, purification, and formulation.

This principle is illustrated by the published diethylstilbestrol (DES) model in estrogen-receptor-positive MCF-7 cells. Although iodine-125 would be expected to have higher decay-specific Auger potency, [^123^I]I-DES produced comparable or even stronger receptor-mediated radiotoxic effects than [^125^I]I-DES. This apparently unexpected finding can be explained by the limited estrogen-receptor capacity of MCF-7 cells and the much higher specific activity achievable with [^123^I]I-DES, which allowed substantially more receptor-associated radioactivity to be delivered to the nuclear fraction. Thus, the DES model shows that experimentally realized radiotoxicity may be governed not only by absorbed dose per nuclear transformation, but also by the amount of activity that can actually be delivered to a finite number of target sites [37].

A different situation applies to iododeoxyuridine. In this model, the radiolabeled nucleoside analogue is not limited by receptor binding but is incorporated into replicating DNA. Therefore, the DNA-proximal source geometry becomes more directly relevant. Consistent with the Goddu/Howell nucleus-to-nucleus comparison, iodine-125 has an approximately 2.2-fold higher cellular S value than iodine-123, closely matching the uncorrected double-strand-break ratio reported for DNA-incorporated [^125^I]IUdR versus [^123^I]IUdR in V79 cells. When repair-permissive decay conditions are considered, this ratio decreases to approximately 1.34, corresponding to 0.45–0.74 double-strand breaks per iodine-123 decay [38].

Thus, the iodine-123/iodine-125 comparison illustrates how physical half-life can influence Auger-emitter radiobiology without altering the absorbed dose per nuclear transformation. For radioiodinated compounds, the shorter half-life of iodine-123 allows substantially higher molar activity than iodine-125, which may become decisive when only a limited number of biologically relevant target sites is available. In receptor-limited systems such as radiolabeled DES derivatives, this higher deliverable target-associated activity can compensate for the lower per-decay Auger potency of iodine-123. In contrast, in DNA-incorporation models such as IUdR, the DNA-proximal source geometry more directly reveals the expected per-decay hierarchy, with iodine-125 showing the higher cellular S value. Therefore, physical half-life should not be treated as an isolated determinant of radiotoxicity, but as a parameter that links radionuclide decay kinetics, achievable molar activity, target-site capacity, subcellular residence, and the experimentally observed biological endpoint.

## 3. Subcellular Localization and Vector-Mediated Auger Radiotoxicity

The preceding sections have considered Auger-emitting radionuclides in terms of decay-associated physicochemical processes and cellular source–target dosimetry. For radiopharmaceutical application, however, the decisive question is where the decay event occurs within the cell.

In this context, the term vector is used in a broad radiopharmaceutical sense and refers to the chemical or biological targeting component of a radiopharmaceutical that transports the radionuclide, ideally after intravenous administration, to a defined cellular target structure. Depending on the nature of this target and the subsequent cellular processing of the radiopharmaceutical, the vector predetermines the binding site, internalization route, intracellular trafficking, retention, and final subcellular localization of the Auger-emitting radionuclide. Thus, the vector does not merely deliver radioactivity to a cell; it defines the source position from which the Auger cascade can act on nearby vulnerable structures.

The Auger cascade itself is governed by atomic physics, but its biological impact is determined by the proximity of the radionuclide to radiosensitive subcellular structures. Nuclear DNA represents the classical high-sensitivity target, as illustrated by DNA-incorporated [^125^I]IUdR. However, biologically relevant Auger-mediated damage is not necessarily restricted to direct DNA incorporation and may also arise from membrane-associated, endosomal, cytoplasmic, mitochondrial, perinuclear, or other intracellular localization patterns. Mitochondria may represent an additional vulnerable subcellular target, particularly when radiopharmaceutical design promotes mitochondrial accumulation and places short-range electron emissions in proximity to mitochondrial membranes or mitochondrial DNA. Recent preclinical work with dual-targeted [^161^Tb]Tb radioconjugates combining a PSMA-binding vector with a mitochondria-tropic triphenylphosphonium moiety demonstrated increased mitochondrial uptake and enhanced radiocytotoxicity compared with [^161^Tb]Tb-PSMA-617 lacking the mitochondrial targeting group. This strategy relies on sequential tumor-cell targeting and subsequent intracellular organelle targeting; the triphenylphosphonium moiety itself is not tumor-specific, and its translational value therefore depends on selective cellular delivery and the avoidance of off-target mitochondrial exposure [39].

### 3.1. Direct DNA Incorporation: [^125^I]IUdR as the Reference Model

Direct incorporation of Auger-emitting radionuclides into DNA represents the most stringent source–target geometry for Auger-mediated radiotoxicity. The prototypical compound is [^125^I]IUdR, a thymidine analogue that is incorporated into DNA during replication and thereby positions iodine-125 directly within the DNA helix, in immediate proximity to neighboring bases and the sugar–phosphate backbone. Under these conditions, the Auger cascade occurs within the molecular environment of chromatin and can generate highly clustered DNA lesions, including complex double-strand breaks [18,28,33,40].

The exceptional potency of DNA-incorporated iodine-125 has been demonstrated across several experimental systems. Early work by Feinendegen established that biologically relevant Auger damage requires decay within, or in immediate proximity to, vital molecular structures, with DNA as the paradigmatic target [18,38]. Subsequent cellular and DNA model studies confirmed that [^125^I]IUdR, once incorporated into mammalian-cell DNA, produces pronounced clonogenic cell killing, cytogenetic damage, and highly localized DNA lesions around the decay site. Repair studies further showed that the resulting double-strand breaks are structurally complex and poorly repaired [33,41,42].

Together, these findings establish direct DNA incorporation as the upper end of Auger-mediated radiotoxicity and emphasize that iodine-125 radiotoxicity is not an intrinsic property of the radionuclide alone, but depends critically on chemical form and subcellular localization. Kassis and co-workers [43] showed that the biological effect of iodine-125 is dominated by the fraction associated with nuclear DNA, whereas extracellular or non-DNA-associated activity contributes far less to reproductive cell death despite contributing to absorbed dose.

Consistent with these observations, our recent work in chemotherapy-sensitive and multidrug-resistant breast cancer cells showed that DNA-localized [^125^I]IUdR induced more pronounced DNA fragmentation than [^125^I]NaI. This supports DNA incorporation as the decisive determinant of radiotoxic efficacy in this model and indicates that DNA-localized Auger emitters may retain activity in drug-resistant tumor cells [44].

Fischer et al. [45] further showed that DNA-directed Auger irradiation can trigger cell-type-dependent biological responses. In human embryonic stem cells and keratinocytes, [^125^I]IUdR induced distinct apoptotic and transcriptional programs, indicating that the outcome of DNA-associated Auger decay depends not only on decay site and chemical form, but also on the differentiation state and intrinsic response capacity of the target cell.

Feinendegen and Neumann [46] reinforced this distinction by comparing DNA-incorporated [^125^I]IUdR with other intranuclear or cellular iodine-125 vectors. Their analysis showed that [^125^I]IUdR was far more toxic than iodine-125 localized in the nucleus without measurable DNA binding. Thus, nuclear localization alone is insufficient; lethal Auger-mediated damage depends on the molecular vector and the exact intranuclear source–target geometry.

Thus, [^125^I]IUdR remains the benchmark for direct DNA-incorporated Auger irradiation, whereas most receptor-targeted radiopharmaceuticals rely on alternative source–target geometries. These are considered in the following sections.

### 3.2. Receptor-Mediated Nuclear and Chromatin-Associated Proximity: Radioestrogen Ligands

Radioestrogen ligands represent a localization strategy distinct from direct DNA incorporation. Unlike [^125^I]IUdR, they are not incorporated into DNA during replication. Their relevance for Auger-emitter therapy derives from estrogen-receptor biology: after intracellular binding, the ligand–receptor complex can translocate to the nucleus, where estrogen receptors act as transcription factors and interact with chromatin through zinc-finger DNA-binding domains at estrogen response elements or through tethering to other DNA-bound transcription factors. In this way, radioestrogens may bring Auger-emitting radionuclides into DNA-proximal chromatin environments without placing the radionuclide directly within the DNA molecule. This concept is supported by early estrogen receptor-directed Auger studies, in which high-specific-activity iodine-123-labeled estrogens showed receptor-specific cytotoxicity in estrogen-receptor-positive cells, whereas estrogen-receptor-negative cells were largely resistant [7,8].

Our own studies followed this mechanistic line. Receptor-directed 16α-[^125^I]iodo-estradiol-3,17β and subsequent radioiodinated diethylstilbestrol derivatives were used to test whether estrogen receptor-mediated localization can translate Auger decay into measurable cellular effects [9,37]. In ER-positive MCF-7 cells, radioiodinated DES showed rapid uptake, predominant nuclear recovery, reduced metabolic viability, and induction of intracellular DNA fragmentation, whereas radioiodide alone produced much weaker or absent DNA-fragmentation effects. These findings support the concept that receptor-mediated intracellular and nuclear-associated localization, rather than exposure to radioiodine per se, determines the radiobiological efficacy of radioestrogen-based Auger-emitter approaches [9,37].

Radioestrogens therefore occupy an instructive middle ground between DNA-incorporated [^125^I]IUdR and conventional receptor-targeted Auger radiopharmaceuticals. They show that direct DNA incorporation is not the only route to biological efficacy, provided that receptor-mediated trafficking brings the radionuclide into DNA-proximal nuclear or chromatin-associated environments.

These findings support the development of future radiometal-chelatable estrogen ligands that use estrogen-receptor binding to deliver the radionuclide to estrogen-receptor-positive cells and promote nuclear or chromatin-associated localization of radioactive decay.

### 3.3. Membrane Binding, Internalization, and Extranuclear Auger Effects

Membrane targeting represents a mechanistically distinct Auger-emitter strategy because the radionuclide is not delivered to DNA or to the nuclear compartment. Paillas et al. [21] showed that iodine-125-labeled monoclonal antibodies bound to cell-surface targets can nevertheless induce cytotoxicity through oxidative stress, membrane-associated signaling, and bystander mechanisms. These findings do not make membrane localization equivalent to direct DNA incorporation; rather, they expand the spectrum of biologically relevant Auger source–target geometries beyond nuclear targeting.

Pouget et al. [20] compared iodine-125 localized at the cell membrane, in the cytoplasm, or in the nucleus. Nuclear targeting produced the strongest cytotoxicity when related to the total number of decays. After normalization to mean nuclear dose, however, membrane-associated iodine-125 was more effective than cytoplasmic iodine-125 and, in SK-OV-3 cells, approached the efficacy of nuclear targeting. This suggests that membrane-associated Auger ionization can be biologically relevant, whereas simple cytoplasmic retention, particularly in lysosomal compartments, may be less effective. This apparent similarity under a specific dose-normalization approach used in that study does not imply radiobiological equivalence between membrane-associated and DNA-proximal irradiation. The relevant molecular targets, damage pathways, dose–response relationships, and relative biological effectiveness may differ substantially.

Behr et al. [47] provided an early in vivo example of this principle. In a colorectal cancer xenograft model, an internalizing iodine-125-labeled antibody produced superior tumor-growth delay compared with the corresponding iodine-131-labeled antibody, whereas this advantage was not observed with a noninternalizing antibody. This supports the view that endocytic uptake can create a more favorable Auger source–target geometry than persistent extracellular or surface-bound localization alone. Endocytic uptake should therefore be understood as altering intracellular trafficking and retention rather than as evidence that endosomal localization itself is radiobiologically equivalent to nuclear or DNA-proximal localization.

#### 3.3.1. Terbium-161 Versus Lutetium-177: Radiobiological Basis for a Potential Advantage

Building on the cellular source–target dosimetry considerations discussed in Section CELLDOSE-Derived Total-Electron Dose Estimates for Terbium-161, terbium-161 should be interpreted primarily as a β^−^/internal-conversion/Auger hybrid radionuclide rather than as a functional analogue of DNA-localized iodine-125. Its most relevant translational comparison is therefore with lutetium-177, not with classical DNA-incorporated Auger emitters. As outlined above, terbium-161 can provide additional local electron dose compared with lutetium-177; however, the biological relevance of this component depends on whether the radiopharmaceutical positions terbium-161 in subcellular compartments where short-range electrons can interact with radiosensitive structures [35,48].

This interpretation does not diminish the physical relevance of terbium-161. Compared with lutetium-177, terbium-161 provides a substantially richer spectrum of short-range electron emissions, including internal-conversion, Auger, and Coster–Kronig electrons. Its potential advantage is therefore physically plausible, but it becomes radiobiologically meaningful only when the labeled vector creates subcellular distributions in which these emissions can deposit dose within effective range of vulnerable cellular structures.

This distinction is particularly important for receptor-targeted radiopharmaceuticals. Unlike [^125^I]IUdR, terbium-161-labeled ligands do not generally generate decay events directly within, or immediately adjacent to, nuclear DNA. Their potential advantage over lutetium-177 is therefore expected to arise from additional short-range electron deposition at the sites reached by the vector, including the cell membrane, endosomal or cytoplasmic compartments, perinuclear regions, or, in selected cases, the nucleus. Thus, the benefit of terbium-161 is intrinsically dependent on vector design, internalization, and subcellular localization [49].

De Nardo et al. [50] extended this comparison to somatostatin analogues and found a dosimetric advantage of terbium-161 over lutetium-177, particularly in small cell clusters and micrometastatic models; biologically, this translated into the possibility of achieving comparable cell damage with lower terbium-161-labeled radiopharmaceutical amounts.

Spoormans et al. [51] further refined this interpretation by showing that the increased nucleus-absorbed dose of terbium-161-labeled somatostatin analogues was mainly attributable to internal-conversion electrons rather than to Auger electrons. At the same time, membrane-associated localization may still allow very short-range Auger electrons to contribute locally to the observed dose response. Terbium-161 should therefore not be regarded simply as a more potent version of lutetium-177, but as a hybrid radionuclide whose biological advantage depends on the subcellular position of the radiopharmaceutical and on the relative contribution of β^−^ particles, internal-conversion electrons, Auger electrons, and Coster–Kronig electrons.

Clinical translation of this concept is already underway, but the clinical evidence should be stated cautiously. An intraindividual pilot dosimetry study comparing [^161^Tb]Tb-PSMA-617 with [^177^Lu]Lu-PSMA-617 in mCRPC reported higher tumor-absorbed doses and a more favorable therapeutic index for [^161^Tb]Tb-PSMA-617, but this was primarily a dosimetric comparison rather than definitive evidence of superior clinical outcome [52]. First-in-human phase 1/2 data with [^161^Tb]Tb-PSMA-I&T indicate feasibility, acceptable safety, and antitumor activity in mCRPC, but these data are single-arm and do not yet establish clinical superiority over [^177^Lu]Lu-PSMA therapy in a randomized setting [53].

Taken together, terbium-161 should not be viewed merely as a lutetium-177 analogue with additional low-energy electron emissions. Rather, it represents a hybrid therapeutic radionuclide that combines β^−^ irradiation with internal-conversion, Auger, and Coster–Kronig electrons. This provides terbium-161 with a mechanistic feature that lutetium-177 does not offer: the capacity to add highly localized short-range electron dose to an established β^−^-therapy platform. Its translational value will therefore depend on radiopharmaceutical design, subcellular trafficking, radiobiological endpoints, and ultimately comparative clinical outcome data [54].

#### 3.3.2. Diagnostic Auger Emitters and the Role of Cellular Localization

The relevance of Auger-emitting radionuclides is not restricted to agents intentionally developed for therapy. Several diagnostic radionuclides, including iodine-123, indium-111, gallium-67, and technetium-99m, also emit Auger, Coster–Kronig, or internal-conversion electrons, as summarized in Table 1. Iodine-123 is not revisited in detail in this section because its radiobiological relevance has already been discussed above in direct comparison with iodine-125, particularly with respect to DNA-associated decay, cellular S values, molar activity, and biologically deliverable activity. For the remaining diagnostic radionuclides considered here, radiobiological relevance depends less on diagnostic use per se than on cellular uptake, intracellular retention, and subcellular localization. Technetium-99m is particularly noteworthy in this regard: although it remains the diagnostic workhorse of nuclear medicine, its short-range electron emissions are rarely considered in routine clinical interpretation.

Häfliger et al. [55] showed that DNA-binding technetium-99m complexes can induce double-strand breaks in plasmid DNA, whereas non-DNA-binding pertechnetate did not produce comparable damage. This principle was subsequently examined in greater detail by the Dresden group. Kotzerke et al. [56] demonstrated that the DNA-binding compound [^99m^Tc]Tc-HYNIC-DAPI produced substantially more plasmid DNA damage than [^99m^Tc]TcO_4_^−^, with damage largely resistant to radical scavenging.

Reissig et al. [16] then refined this concept using [^99m^Tc]Tc-labeled pyrene derivatives with different spacer lengths between the DNA-intercalating pyrene moiety and the technetium-99m core. Closely DNA-associated derivatives induced single- and double-strand breaks, whereas increasing the distance markedly reduced direct DNA damage.

Related work with acridine orange-containing technetium-99m tricarbonyl complexes further demonstrated that molecular design can promote DNA intercalation, nuclear retention, and measurable radiobiological effects. Studies in plasmid DNA and prostate cancer cells showed that these constructs can induce DNA damage, with the magnitude of the effect depending strongly on the distance between the technetium-99m core and the DNA double helix [57,58].

Together, these studies show that technetium-99m can cause measurable DNA damage when placed in close DNA proximity, while unbound pertechnetate acts predominantly through indirect radical-mediated mechanisms.

Indium-111 provides another instructive example of a diagnostic radionuclide with Auger-electron relevance under appropriate source–target conditions. Sahu et al. [59] showed that indium-111 decay can induce strand breaks in pBR322 plasmid DNA, with the biological effect depending on whether the radionuclide is DNA-bound or present in solution.

In cellular targeting studies, Cai et al. [60] showed that the response to [^111^In]In-DTPA-hEGF in breast cancer cells was linked to EGFR expression, nuclear uptake, γH2AX induction, and clonogenic survival. This supports the concept that receptor-mediated internalization and nuclear translocation can make indium-111 decay biologically consequential.

Similarly, Costantini et al. [61] showed that modification of [^111^In]In-labeled trastuzumab with nuclear localization sequences enhanced nuclear routing and cytotoxicity in HER2/neu-positive breast cancer cells. Thus, indium-111 illustrates how a diagnostic radionuclide can acquire therapeutic relevance when molecular design promotes cellular uptake, intracellular trafficking, and nuclear proximity.

Gallium-67 has also been re-evaluated as a therapeutically relevant Auger emitter. Othman et al. [62] argued that gallium-67 had been largely neglected as a therapeutic radionuclide and compared its potential with that of indium-111. In subsequent experimental work, they showed that [^67^Ga]Ga-THP-trastuzumab reduced viability and clonogenic survival in HER2-positive breast cancer cells when the radiopharmaceutical was cell-bound [63].

These examples should not be interpreted as a general radiotoxic warning against established diagnostic radiopharmaceuticals. The decisive condition for biologically relevant Auger toxicity is not diagnostic use per se, but nanoscale proximity to radiosensitive intracellular targets. In most routine imaging applications, technetium-99m, indium-111, gallium-67, or iodine-123 are administered at diagnostic activities and are not retained in direct DNA or nuclear proximity. Under these conditions, their Auger component is usually of limited radiobiological consequence.

The situation changes when these radionuclides are deliberately attached to molecules that promote cellular uptake, intracellular retention, nuclear routing, DNA binding, or very short source–target distances. Only then can their Auger, Coster–Kronig, or internal-conversion electrons produce measurable biological effects, become toxicologically relevant, or be exploited therapeutically. Thus, diagnostic Auger emitters highlight a central rule of Auger radiobiology: the decay scheme creates the physical possibility of localized damage, but molecular design and subcellular localization determine whether this possibility remains negligible, becomes measurable, or can be exploited therapeutically.

## 4. Conclusions

Auger-emitting radionuclides occupy a unique position in radiopharmaceutical research because their biological relevance is determined at the interface of radionuclide decay, atomic and molecular relaxation, cellular trafficking, and subcellular dosimetry. Their therapeutic potential cannot be inferred from the decay scheme alone. The decisive question is not only how many Auger or Coster–Kronig electrons are emitted per decay, but where, when, and in which molecular environment the decay occurs.

DNA-incorporated [^125^I]IUdR remains the most stringent reference model for Auger-mediated radiotoxicity, illustrating the upper limit of biological effectiveness when decay takes place within nanometer distance of the DNA backbone. However, this model should not be generalized uncritically to all Auger-emitting radiopharmaceuticals. Most targeted radiopharmaceuticals do not enter chromatin, and their effects must therefore be interpreted through alternative source–target geometries, including membrane-associated irradiation, endosomal or lysosomal retention, cytoplasmic or perinuclear localization, and oxidative stress- or bystander-mediated mechanisms.

Cellular S values provide an important quantitative framework for comparing radionuclides under defined source–target assumptions, but they are not a direct ranking of therapeutic efficacy. Biological outcome also depends on physical half-life, dose rate, specific activity, ligand pharmacokinetics, intracellular retention, repair kinetics, proliferation, and the number of available molecular binding sites. The comparison between iodine-123 and iodine-125, as well as the emerging role of terbium-161, illustrates that the biological value of an Auger or internal-conversion electron emitter is intrinsically vector-, time-, and localization-dependent.

This principle also applies to diagnostic radionuclides such as technetium-99m, indium-111, gallium-67, and iodine-123. These radionuclides are not biologically irrelevant merely because they are used for imaging, but their diagnostic use does not imply general radiotoxic concern. Their biological significance is conditional: if they are rapidly cleared or remain distant from sensitive subcellular targets, the Auger component is usually of limited consequence; if they are internalized, retained, or brought into nanoscale proximity to DNA, chromatin, membranes, mitochondria, or other vulnerable structures, they may become biologically active nanoscale radiation sources.

The central message of this review is that Auger radiopharmaceuticals must be designed and interpreted as integrated molecular systems. The radionuclide defines the physical possibility of localized energy deposition, whereas the vector molecule determines whether this possibility is biologically realized. Future progress will depend on radiopharmaceutical designs that combine favorable emission characteristics with controlled intracellular trafficking, experimentally verified subcellular localization, cellular dosimetry, and biologically meaningful endpoints. In this sense, Auger-emitter radiopharmaceuticals are not simply another class of therapeutic radionuclides; they represent a test case for whether nuclear medicine can deliberately exploit radiation effects at the molecular scale.

A particularly promising future direction will be the development of radiopharmaceutical vectors that exploit biological features shared by multiple tumor entities rather than single tumor-type-specific markers. Such vectors would be especially valuable if they combine broad tumor recognition with intracellular trafficking toward radiosensitive subcellular regions, including perinuclear or chromatin-associated environments. In this setting, Auger- and internal-conversion-electron emitters could be used not merely as radionuclide labels, but as molecularly positioned sources of highly localized radiation damage. Although this concept remains experimentally demanding, it may represent one of the most attractive routes toward broadly applicable Auger-based radiopharmaceutical therapy.

## Figures and Tables

**Table 1:** Physical emission characteristics and cellular S values for selected Auger-electron-emitting radionuclides and β^−^-emitting comparator radionuclides.

RN	T_1_/_2_	Decay	n_AE/CK_	E_AE/CK_ (keV)	n_IC_	E_IC_ (keV)	S (C←C)	S (C←CS)	S (N←N)	S (N←Cy)	S (N←CS)	S_rel_
**Auger/Coster–Kronig and internal-conversion electron emitters**												
**I-125**	59.4 d	EC	23.0	12.0	0.9	7.3	9.58 × 10^−4^	5.20 × 10^−4^	3.54 × 10^−3^	2.01 × 10^−4^	1.14 × 10^−4^	1.00
**I-123**	13 h	EC	13.7	7.2	0.2	21.0	6.29 × 10^−4^	3.43 × 10^−4^	1.59 × 10^−3^	1.31 × 10^−4^	7.87 × 10^−5^	0.45
**In-111**	67 h	EC	7.4	6.9	0.2	27.9	6.02 × 10^−4^	3.33 × 10^−4^	1.48 × 10^−3^	1.65 × 10^−4^	1.07 × 10^−4^	0.42
**Ga-67**	78 h	EC	5.0	6.6	0.3	29.7	7.43 × 10^−4^	3.96 × 10^−4^	1.90 × 10^−3^	2.01 × 10^−4^	4.54 × 10^−5^	0.54
**Tc-99m**	6 h	IT	4.4	0.9	1.1	15.2	3.16 × 10^−4^	1.65 × 10^−4^	8.33 × 10^−4^	4.00 × 10^−5^	2.24 × 10^−5^	0.24
**Tl-201**	73 h	EC	20.9	14.8	0.9	29.9	1.59 × 10^−3^	8.57 × 10^−4^	4.03 × 10^−3^	4.97 × 10^−4^	1.87 × 10^−4^	1.14
**Pt-195m**	4.0 d	IT	36.6	23.1	2.8	161.4	3.87 × 10^−3^	2.25 × 10^−3^	8.89 × 10^−3^	1.93 × 10^−3^	1.07 × 10^−3^	2.51
**Pt-193m**	4.3 d	IT	27.4	10.9	3.0	126.8	2.32 × 10^−3^	1.26 × 10^−3^	5.83 × 10^−3^	8.40 × 10^−4^	2.91 × 10^−4^	1.65
**β^−^-emitting comparator radionuclides**												
**I-131**	8.02 d	β^−^	n.a.	n.a.	n.a.	n.a.	3.18 × 10^−4^	2.04 × 10^−4^	6.57 × 10^−4^	2.08 × 10^−4^	1.38 × 10^−4^	0.19
**Lu-177**	6.65 d	β^−^	n.a.	n.a.	n.a.	n.a.	4.72 × 10^−4^	2.93 × 10^−4^	1.02 × 10^−3^	2.78 × 10^−4^	1.72 × 10^−4^	0.29

Notes: Physical emission data for Auger/Coster–Kronig and internal-conversion electrons are taken from Ku et al. [12]; cellular S values are based on the Goddu/Howell cellular S-value framework for a spherical cell model with a cell radius of 7 µm and a nucleus radius of 5 µm [30]. The numerical S values and the resulting relative relationships are specific to these cell and nucleus dimensions and may vary with cellular geometry and source distribution. S values are given in Gy·Bq^−1^·s^−1^; C, whole cell; CS, cell surface; N, nucleus; Cy, cytoplasm. Decay denotes the main decay mode; n_AE/CK_, mean number of Auger/Coster–Kronig electrons emitted per decay; E_AE/CK_, total Auger/Coster–Kronig electron energy emitted per decay in keV; n_IC_, mean number of internal-conversion electrons emitted per decay; E_IC_, total internal-conversion electron energy emitted per decay in keV. S(T←S) denotes the absorbed dose to target region T per unit cumulated activity in source region S. Accordingly, S(C←C) represents whole-cell self-dose, S(C←CS) dose to the whole cell from activity at the cell surface, S(N←N) nucleus-to-nucleus self-dose, S(N←Cy) dose to the nucleus from cytoplasmic activity, and S(N←CS) dose to the nucleus from activity at the cell surface. S_rel_ denotes S(N←N) normalized to the corresponding S(N←N) value of I-125; thus, I-125 is assigned a value of 1.00. I-131 and Lu-177 are included as β^−^-emitting comparator radionuclides. n.a., not applicable.

## Data Availability

No new data were created or analyzed in this study. Data sharing is not applicable to this article.
